# Inhibition of Breast Cancer Metastasis Suppressor 1 Promotes a Mesenchymal Phenotype in Lung Epithelial Cells That Express Oncogenic K-Ras^V12^ and Loss of p53

**DOI:** 10.1371/journal.pone.0095869

**Published:** 2014-04-24

**Authors:** Emily H. Hall, Yuan Liu, Aizhen Xiao, Lisa Shock, David L. Brautigan, Marty W. Mayo, Prasad S. Adusumilli, David R. Jones

**Affiliations:** 1 Department of Surgery, University of Virginia, Charlottesville, Virginia, United States of America; 2 Department of Biochemistry and Molecular Genetics, University of Virginia, Charlottesville, Virginia, United States of America; 3 Department of Microbiology, Immunology &Cancer Biology, and Center for Cell signaling, University of Virginia, Charlottesville, Virginia, United States of America; 4 Department of Surgery, Memorial Sloan-Kettering Cancer Center, New York, New York, United States of America; Institute of Experimental Endocrinology and Oncology ‘G. Salvatore’ (IEOS), Italy

## Abstract

Expression of the breast cancer metastasis suppressor 1 (BRMS1) protein is dramatically reduced in non-small cell lung cancer (NSCLC) cells and in primary human tumors. Although BRMS1 is a known suppressor of metastasis, the mechanisms through which BRMS1 functions to regulate cell migration and invasion in response to specific NSCLC driver mutations are poorly understood. To experimentally address this, we utilized immortalized human bronchial epithelial cells in which p53 was knocked down in the presence of oncogenic K-Ras^V12^ (HBEC3-p53KD-K-Ras^V12^). These genetic alterations are commonly found in NSCLC and are associated with a poor prognosis. To determine the importance of BRMS1 for cytoskeletal function, cell migration and invasion in our model system we stably knocked down *BRMS1*. Here, we report that loss of BRMS1 in HBEC3-p53KD-K-Ras^V12^ cells results in a dramatic increase in cell migration and invasion compared to controls that expressed BRMS1. Moreover, the loss of BRMS1 resulted in additional morphological changes including F-actin re-distribution, paxillin accumulation at the leading edge of the lamellapodium, and cellular shape changes resembling mesenchymal phenotypes. Importantly, re-expression of BRMS1 restores, in part, cell migration and invasion; however it does not fully reestablish the epithelial phenotype. These finding suggests that loss of *BRMS1* results in a permanent, largely irreversible, mesenchymal phenotype associated with increased cell migration and invasion. Collectively, in NSCLC cells without p53 and expression of oncogenic K-Ras our study identifies BRMS1 as a key regulator required to maintain a cellular morphology and cytoskeletal architecture consistent with an epithelial phenotype.

## Introduction

Lung cancer has the highest mortality rate among cancers affecting both men and women in the United States with an overall survival rate of 15% [1]. The overwhelming cause of death following a diagnosis of lung cancer is the development of metastatic disease. Metastasis is a multi-step process that includes local invasion, intravasation, survival in circulation, extravasation, and ultimately proliferation of micrometastases [2]. Metastasis suppressor genes inhibit the formation and development of metastases without affecting primary tumor growth. This class of proteins is recognized for their ability to inhibit steps along in the metastatic cascade [3].

Breast cancer metastasis suppressor 1 (*BRMS1*) is a metastasis suppressor gene that is conserved across species and has been shown to decrease the development of metastasis in lung, breast, melanoma, bladder, and ovarian malignancies [4]–[7]. We have shown that BRMS1 mRNA and protein levels are significantly reduced in non-small cell lung cancer (NSCLC) cell lines and human NSCLC tumors [8]. Our group has also shown that in response to inflammatory signals RelA/p65 transcription factor recruits DNMT1 to DNA resulting in promoter methylation and transcriptional silencing of the *BRMS1* promoter [9], [10]. This is highly relevant because loss of the *BRMS1* allele correlates with decreased survival in patients with NSCLC [5]. BRMS1 functions as a co-repressor in the mSin3A complex [8], [11] and modulates the downstream effectors of metastases including CXCR4 [12], miRNAs [13], and osteopontin [14]. Recently, we have shown that BRMS1 has a unique E3 ligase function resulting in degradation of the histone acetyltransferase p300. Mutation of the E3 ligase CLD motif in BRMS1 resulted in a significant increase in lung cancer metastasis in a lung cancer mouse model [15].

We hypothesize that BRMS1 is a primary inhibitor of cell migration and invasion in NSCLC. The majority of studies investigating proteins and signal transduction pathways that modulate cancer metastases have used cancer cell lines and clinical tumor samples. While important, use of these model systems to examine the specific effects of a singular gene or protein on the metastatic process is a significant limitation given that there are numerous pro-metastatic proteins and processes that are dysregulated. To experimentally address this limitation and to examine the BRMS1 specific effects in regulating cell migration and invasion, we chose to exploit two established genetic alterations observed in human NSCLC - the loss of the p53 tumor suppressor and gain-of function mutation in the *K-Ras* allele [16]. To better understand the functional consequence of these two genetic alterations Sato and colleagues knocked down p53 and/or introduced oncogenic K-Ras into immortalized human bronchial epithelial HBEC3 cells (HBEC3-p53KD-K-Ras^V12^). While HBEC3-p53KD-K-Ras^V12^ cells partially progressed toward a malignant phenotype, these alterations failed to confer a full malignant phenotype [17]. Thus, HBEC3-p53KD-K-Ras^V12^ cells offered an excellent model system to examine the importance at several levels of BRMS1 in inhibiting cellular processes involved in metastasis. First, the genetic alterations that result in immortalization and pre-malignancy for HBEC3-p53KD-K-Ras^V12^ cells are known, and second, HBEC3-p53KD-K-Ras^V12^ cells express BRMS1 protein at comparable levels to the immortalized HBEC3 cells. We chose this defined genetic background because alterations of p53 are seen in 50% of NSCLC adenocarcinoma histologies, whereas, oncogenic K-Ras is seen in 30% of adenocarcinoma[16]. We specifically chose the combined p53KD and oncogenic K-Ras genetic background given that loss of p53 combined with K-Ras mutations in human NSCLC is associated with a more aggressive malignancy [18].

In this study we analyzed the importance of BRMS1 in regulating migration, invasion, and actin cytoskeletal signaling by knocking down *BRMS1* in HBEC3 p53KD-K-Ras^V12^ cells. We report that loss of *BRMS1* results in increased cell migration, invasion, alterations in the actin cytoskeletal network, and overall changes in cell morphology consistent with mesenchymal-like migratory phenotypes. These results indicate that BRMS1 functions as a metastasis suppressor to inhibit inappropriate cellular and morphological changes that promote invasion and intravasation. Importantly, BRMS1 inhibits these cellular phenotypes even in the context of oncogenic K-ras expression and loss of p53 tumor suppressor activity, suggesting that intratumoral BRMS1 expression needs to be down-regulated in NSCLC to fulfill cellular phenotypes known to promote metastasis.

## Materials and Methods

### Cell Culture and Reagents

The HBEC3 cells (Control, p53KD, K-Ras^v12^ and HBEC3-p53KD-K-Ras^v12^) were a generous gift from John D. Minna at the University of Texas Southwestern Medical Center [17]. The HBEC3 cells were immortalized using mouse Cdk4 and hTERT and were maintained in Keratinocyte-SFM (Life Technologies, Grand Island, NY) medium containing 50 µg/mL bovine pituitary extract and 5 ng/mL EGF (Life Technologies). HEK293T cells and NSCLC cell lines (H1975, H1299, A549, H358 and Calu-3) were purchased from ATCC. HEK293T cells were maintained in DMEM with 10% FBS. NSCLC H1975, H1299, A549 and H358 cells were grown in RPMI with 10% FBS. NSCLC Calu-3 cells were maintained in Eagle's Minimum Essential Medium (ATCC) with 10% FBS. Plasmids encoding shRNA BRMS1 or shRNA control were created by inserting the human *BRMS1* sequence 5′-gtacatgcttcaagagatc-3′ or a scramble DNA sequence respectively [19] into HpaI-XhoI of the lentiviral pSicoR vector (Addgene, Cambridge, MA). The virus packaging plasmids pMDLg/pRRE, pRSV-Rev and pMD2.G were purchased from Addgene. Primary antibodies used in this study include: p53 and actin (Santa Cruz Biotechnology, Santa Cruz, CA), K-Ras (Abgent, San Diego, CA), BRMS1 (Abcam, Cambridge, MA), and paxillin (Cell Signaling, Danvers, MA). Adenoviral-Cre recombinase (Ad-Cre) was purchased from the University of Iowa (Iowa City, IA).

### Virus Generation and Infection

Lentiviruses were generated as described previously [20]. Briefly, HEK 293T cells were plated at 50% confluence the day before transfection. The next day cells were co-transfected with 10 µg lentiviral pSicoR plasmid encoding shRNA BRMS1 or shRNA control and 5 µg each of the packaging plasmid DNA (pMDLg/pRRE, pRSV-Rev and pMD2.G) using transfection reagent Polyfect (Qiagen, Valencia, CA). Mixed supernatants were collected at 36 h and 60 h after transfection, filtered through a 0.4-µm filter and used to infect HBEC3 cells.

HBEC3 cells or NSCLC cells were plated into 100 mm culture dishes at 50% confluence the day before infection. The next day the first round of infections was performed by adding viruses contained media 3 ml with polybrene (8 µg/ml, Santa Cruz Biotechnology) per dish. The second round of infection was repeated 8 h apart. GFP-positive cells were sorted by flow cytometry at 4 days post-infection. Single GFP-positive cells were expanded and screened for the BRMS1 expression. The clones with more than 80% of *BRMS1* knockdown were selected.

### Immunoblot Analysis

Proteins from HBEC3 cell lysates were separated on 10% SDS-PAGE. Membranes were incubated with primary antibodies at a concentration of 1∶1000 and secondary antibodies (Santa Cruz Biotechnology) at a concentration of 1∶5000. Immunoblots were incubated with Super Signal West Pico Chemiluminescent Substrate (Thermo Scientific) and visualized by exposure to film.

### Cell Migration and Cell Invasion Assays

Cells were seeded into the top portion of cell culture inserts with 8.0 µm pores (BD Biosciences, San Jose, CA) at a density of 25,000 cells in serum free media, in duplicate. For cell migration assays the inserts were not coated, for the invasion assays, the inserts were coated with type IV collagen at a concentration of 0.25 mg/ml. The bottom portion of the wells included 1% FBS-media. Cells were allowed to migrate for 6 hours or invade for 22 hours. At the specified time point, inserts were fixed and stained with crystal violet. The inserts were viewed with an Olympus SZX12 low magnification microscope (Olympus America Inc., Lehigh Valley, PA). Images were captured with an Olympus DP70 digital camera and analyzed with the DP Manager software. Cells that were present on the underside of the filters of the transwell assay were counted.

### Ad-Cre treatment of cells

Cells were washed with PBS and incubated with or without Ad-Cre at a concentration of 100∶1 MOI in 2% serum-media for four hours, media containing 10% FBS was added to cells and they were allowed to grow for additional 24 hours. At this point, cell migration, invasion, immunofluorescence and immunoblot experiments were carried out as described.

### Immunofluorescence

After specified treatments, cells were seeded into 4-well glass chamber slides at a density of 25,000 cells per well and grown overnight. The next day cells were fixed with 3.7% formaldehyde-PBS at RT for 15 minutes, washed with PBS, permeabilized with 0.01% triton-PBS for 5 minutes at RT, and then washed and blocked with 1% BSA-PBS for thirty minutes at RT. Cells were incubated with an anti-paxillin antibody (1∶250 in 1% BSA-PBS) for 1 hr at RT. Wells were incubated with an Alexa Fluor 568 secondary antibodies (Life Technologies) for 1 hr at RT at a concentration of 1∶2000 in 1% BSA-PBS. Wells were washed and cover slips were mounted using UltraCruz Mounting Medium containing DAPI (Santa Cruz Biotechnology). Slides were visualized by a HBO lamp with dichroic filters for imaging DAPI, FITC, and TRITC on an Olympus BX51 high magnification microscope (Olympus America Inc.). Images were captured with an Olympus DP70 digital camera and analyzed with the DP Manager software. When analyzing F-actin, cells were seeded, grown, and fixed as described above. At that point, cells were incubated with Rhodamine-Phalloidin (Life Technologies) at a concentration of 5 units for thirty minutes at RT. Cells were washed and imaged as described above. The shape factor and angle of the leading edge of 30 cells in each group were measured using the ImageJ program [19].

Cell shape factor was calculated using F-actin stained cells and the formula, S = 4πA/p2, where A =  the area of the cell and P =  the perimeter of the cell. A cell shape score of 1 is a round cell with little motility and a score of less than 1 represents a more elongated cell shape with increased motility [21].

### Scratch Wound Assay

After specified treatments, cells were seeded into 4-well glass chamber slides at a density of 100,000 cells per well. The next day, cellular monolayers were scratched using sterile tissue culture scrapers and 6 hours post-scraping cells were fixed and stained as described above. The migration of cells toward the wound was analyzed using ImageJ software (http://rsb.info.nih.gov) and expressed as percentage of wound closure as described previously [22]

### Murine xenograft model

All animal experiments were approved by the University of Virginia Animal Care and Use Committee (Protocol number: 3765). The flanks of 6 week old athymic nude mice (NCI, Frederick, MD) were inoculated with 0.1 ml of serum and antibiotic free media containing 2×10^6^ cells of HBEC3-p53KD-K-Ras^v12^ or HBEC3-p53KD-K-Ras^v12^-BRMS1KD for each group (N = 10). Mice were maintained under standard animal husbandry conditions. Mice were analyzed twice a week for tumor growth.

### Statistical Analysis

The results of all experiments represent the mean ± standard deviation (S.D.) of three independent experiments performed in duplicate or triplicate. Statistical significance between control and BRMS1 groups were determined by two tailed, paired Student's t-test. A p-value of <0.05 was considered significant.

## Results

### BRMS1 KD increases cell migration and invasion in p53 deficient and K-Ras^V12^ HBEC3 cells

**Figure 1:**
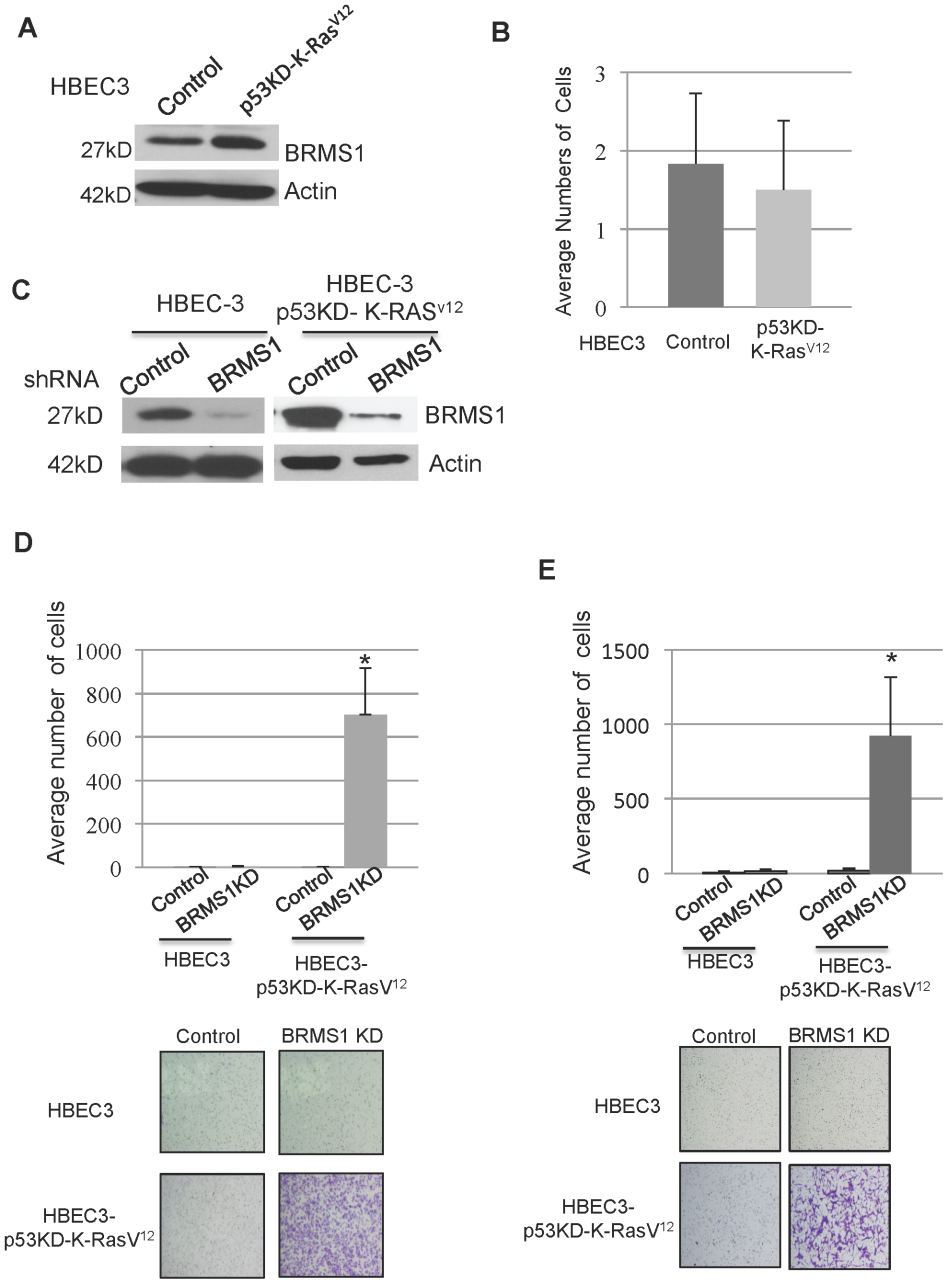
BRMS1 KD significantly increases cell migration and invasion in HBEC3 cells with p53KD and oncogenic K-Ras. A) Immunoblot analysis was performed to access the protein levels of BRMS1 in HBEC3 and HBEC3-p53KD-K-Ras^v12^ cells. Actin is used as a loading control. B) Cell migration assays were conducted using HBEC3 and HBEC3-p53KD-K-Ras^v12^ cells. Graphical representation of the mean ±S.D. of three independent experiments. C) HBEC3 and HBEC3-p53KD-K-Ras^v12^ cells were infected lentiviruses encoding shRNA BRMS1 or shRNA control. The protein levels of BRMS1 were probed by immunoblots. Actin is used as a loading control. D) HBEC3 and HBEC3-p53KD-K-Ras^v12^ cells were infected lentiviruses encoding shRNA BRMS1 (BRMS1 KD) or shRNA control (Control). Cell migration assays were performed. Graphical representation of the mean ±S.D. of three independent experiments and * p≤0.05 compared to control of the same cell line. E) HBEC3 and HBEC3-p53KD-K-Ras^v12^ cells were infected lentiviruses encoding BRMS1 KD or Control. Cell invasion assays were performed and invaded cells were counted under microscope with 10X magnification. Graphical representation of the mean ±S.D. of three independent experiments and * p≤0.05 compared to control of the same cell line.

To initiate experiments we first confirmed by immunoblot analysis that HBEC3-p53KD-K-Ras^V12^ cells expressed BRMS1 protein compared to control HBEC3 cells (Fig. 1A). Moreover, as previously reported [17], HBEC3-p53KD-K-Ras^V12^ cells displayed a loss of p53 and expressed oncogenic K-Ras^V12^ protein, compared to HBEC3 cells (Fig. S1A). Finally, consistent with previous findings [17], we observed non-significant changes in cell migration between HBEC3-p53KD-K-Ras^V12^ and HBEC3 control cells (Fig. 1B).

To examine the specific contributions of BRMS1 to cellular processes responsible for metastasis we stably knocked down *BRMS1* (BRMS1-KD) in HBEC3 cells (Fig. 1C and Fig. S1B). As shown in Fig. 1D and 1E, the knockdown of BRMS1 expression in HBEC3-p53KD-K- Ras^V12^ cells resulted in a significant increase in both migration (>650-fold difference) and cell invasion (>800-fold difference) compared to HBEC3-p53KD-K-Ras^V12^ cells in which BRMS1 is fully expressed. As expected, the knockdown of BRMS1 had no effect on cellular migration and invasion of control or K- Ras^V12^ HBEC3 cells (Figs. 1D, 1E, and Figs. S1C, S1D). Knockdown of BRMS1 did enhance the migration but not their invasion ability of p53 KD HBEC3 cells, (Figs. S1C and D). Since knockdown of BRMS1 expression failed to affect both migration and invasion of immortalized HBEC3 cells (control, p53 KD, and K-Ras^V12)^, these results strongly suggests that loss of BRMS1 controls cell migration and invasion in response to genomic alterations such as LOH of p53 and the generation of oncogenic K-Ras.

### Re-expression of BRMS1 partially restores non-invasive cell phenotypes

**Figure 2:**
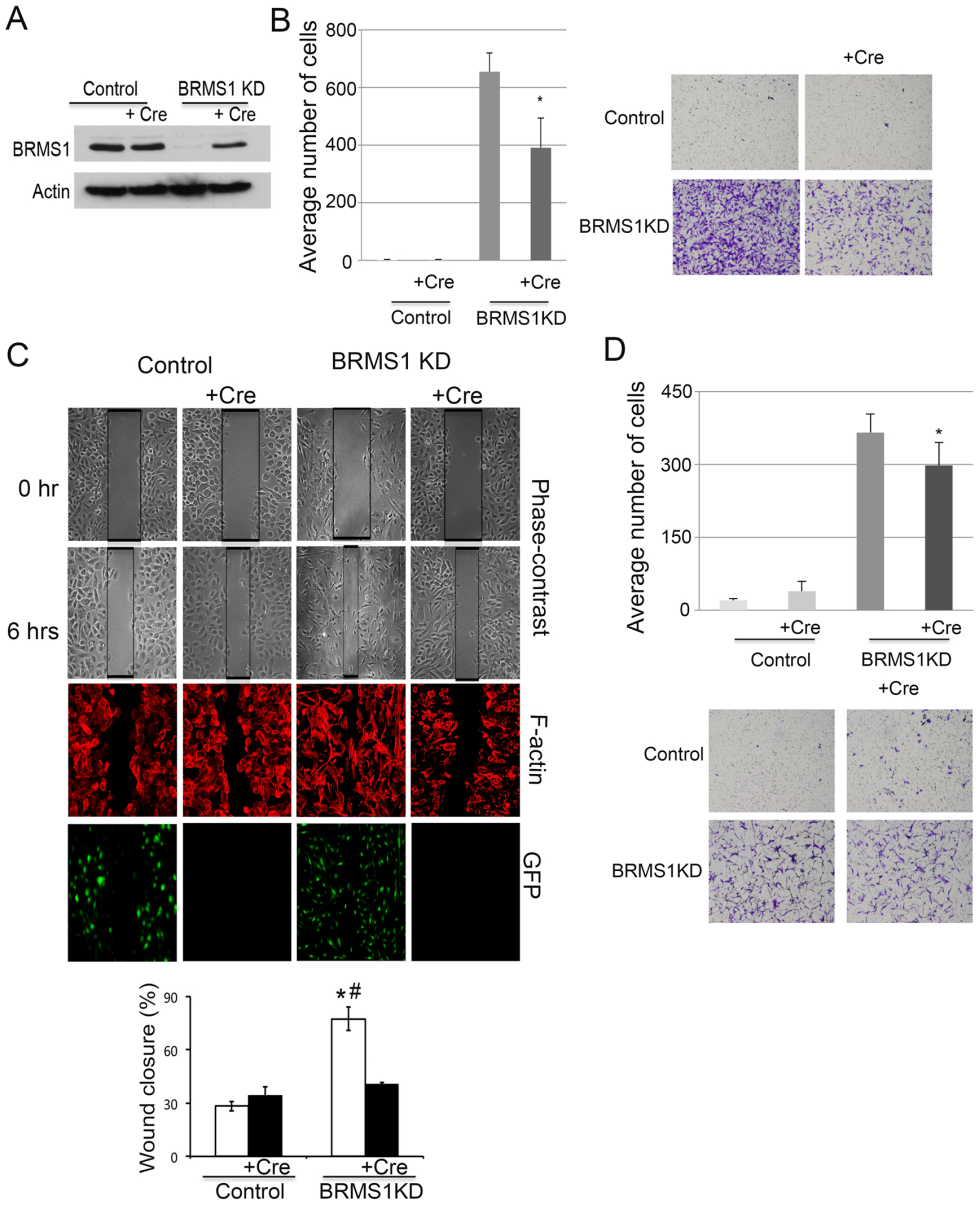
Re-expression of BRMS1 reduces cell migration and invasion. A) HBEC3-p53KD-K-Ras^v12^ Control and BRMS1 KD cells were treated with or without Ad-Cre. The protein levels of BRMS1 were assessed by Western blots. Actin is used as a loading control. B) HBEC3-p53KD-K-Ras^v12^ Control and BRMS1 KD cells were treated with or without Ad-Cre. Cell migration assays were performed. Graphical representation of the mean ±S.D. of three independent experiments and * p≤0.05 compared to control of the same cell line. C) Scratch wound assays with HBEC3-p53KD-K-Ras^v12^ Control and BRMS1 KD cells with and without Ad-Cre treatment. The photographs show the phase-contrast scratch wounds or stained with rhodamine-phalloidin. GFP expression is shown as a control for the efficiency of Ad-Cre. The bar graph shows percentage of wound closure. * p≤0.01 compared to control and # p≤0.01 compared to BRMS1 KD with Cre. D) HBEC3-p53KD-K-Ras^v12^ Control and BRMS1 KD cells were treated with or without Ad-Cre. Cell invasion assays were performed and invaded cells were counted under microscope with 10X magnification. Graphical representation of the mean ±S.D. of three independent experiments and * p≤0.05 compared to control of the same cell line.

Given that the knockdown of *BRMS1* in HBEC3-p53KD-Ras^V12^ cells dramatically increased cell migration, we next wanted to confirm these effects were specific to the loss of *BRMS1* expression. To address this HBEC3-p53KD-K-Ras^V12^ cells stably expressing shRNA BRMS1 were treated with replication-defective adenovirus encoding the Cre recombinase (Ad-Cre). Since the shRNA targeting *BRMS1* are flanked by LoxP sites, this enabled us to treat cells with Ad-Cre resulting in excision of the RNAi in HBEC3-p53KD-Ras^V12^ cells. Following exposure of cells to Ad-Cre recombinase, immunoblots confirmed re-expression of BRMS1 in the HBEC3-p53KD-K-Ras^V12^ -BRMS1 KD cells, compared to HBEC3-p53KD-K-Ras^V12^ control cells (Fig. 2A). Importantly, re-expression of BRMS1 significantly decreased cell migration and invasion compared to HBEC3-p53KD-K-Ras^V12^ -BRMS1 KD cells that did not receive the Cre recombinase in migration and invasion chamber assays, as well as scratch wound assays (Figs. 2B, C and D). Collectively, data shown in Figs. 1 and 2 indicate that the loss of BRMS1 greatly potentiates cell migration and invasion in HBEC3-p53KD-K-Ras^V12^ cells, while concomitantly demonstrating that cell migration profiles in BRMS1-KD cells are not fully restored following Ad-Cre-mediated re-expression of BRMS1.

### BRMS1 KD in HBEC3 p53KD and Kras^v12^ cells do not form *in vivo* tumors

While Soto et al. had previously shown that combined oncogenic changes of p53KD and Kras^v12^ in HBEC3 cells were insufficient to confer a full malignant phenotype in a mouse model [17], we asked whether the loss of the metastasis suppressor BRMS1 may result in tumor formation. However, when the HBEC3-p53KD-Kras^v12^-BRMS1 KD cells were injected into the flank of nude mice, no tumors were found over a 3 month period of observation (data not shown). This data indicates that BRMS1 KD in HBEC3 cells is insufficient to confer a full malignant phenotype even in the presence of an oncogenic background.

### BRMS1 is involved in actin arrangement and paxillin redistribution

During the characterization of HBEC3-p53KD-K-Ras^V12^ cells we observed a profound change in cell morphology in BRMS1-KD cells. While HBEC3-p53KD-K-Ras^V12^ cells had a typical epithelial cobblestone appearance, cells in which *BRMS1* was silenced exhibited a more mesenchymal spindle-shaped, elongated morphology (Fig. S2A). Importantly, this change in cell shape was not observed in immortalized HBEC3 cells following the loss of BRMS1 expression (Fig. S2B), suggesting that the changes observed in HBEC3-p53KD-K-Ras^V12^ cells were due to the genetic makeup of these cells.

**Figure 3:**
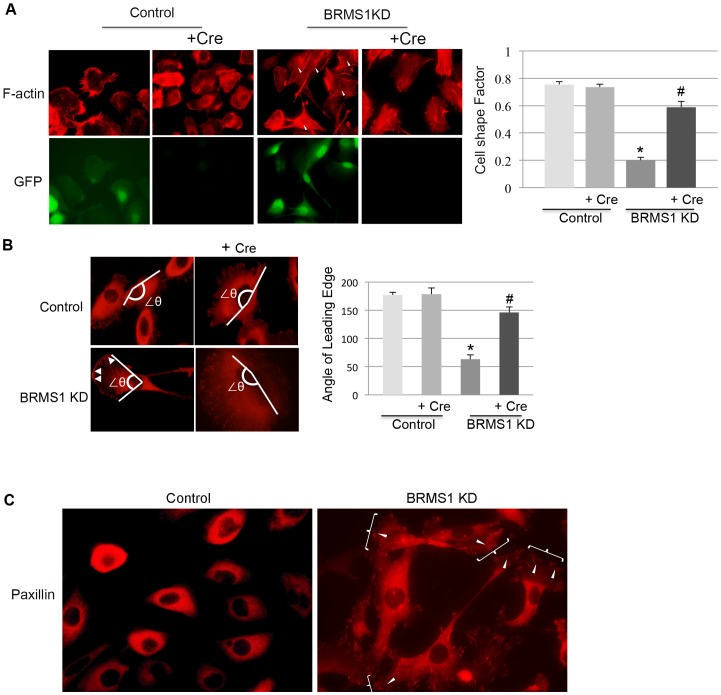
BRMS1 KD regulates actin arrangement and paxillin distribution. A) HBEC3-p53KD-K-Ras^v12^ Control and BRMS1 KD cells were treated with or without Ad-Cre. F-actin was visualized by Rhodamine-Palloidin staining. GFP expression is shown as a control for the efficiency of Ad-Cre. The arrows indicate stress fibers. The shape factor was calculated, * p≤0.05 compared to the no treatment group in control cell line, # p≤0.05 compared to no treatment group in the same cell line. B) HBEC3-p53KD-K-Ras^v12^ Control and BRMS1 KD cells were treated with or without Ad-Cre. The cells were stained with paxillin. The white arrows indicate the accumulation of paxillin at the leading edge of cell. ∠θ represented the span of angle of the leading edge, * p≤0.05 compared to no treatment group in control cell line, # p≤0.05 compared to no treatment group in the same cell line. C) The distribution of paxillin was visualized by immunofluorescence in HBEC3-p53KD-K-Ras^v12^ Control and BRMS1 KD cells. The arrows indicate the accumulation of paxillin at the leading edge of cell and the “}”indicate the lamellapodia.

Since polymerization and depolymerization of filamentous actin (F-actin) controls cytoskeletal reorganization associated with cell morphology and motility [23], we hypothesized that the knockdown of BRMS1 would impact actin reorganization. As shown in Fig. 3A and Fig. S2C, the knockdown of BRMS1 increased the formation of filopodia, lamellapodia and stress fibers in HBEC3-p53KD-K-Ras^V12^ cells. This cellular phenotype was partially reverted following treatment with Ad-Cre-recombinase to induce BRMS1 re-expression (Fig. 3A and Fig. S2C). The efficiency of Ad-Cre is apparent by the loss of GFP expression (Figs. 3A and Figs. S2C and S2D). The loss of BRMS1 expression in HBEC3-p53KD-K-Ras^V12^ cells resulted in a significantly lower shape factor score (0.20 vs. 0.75, p<0.05) compared to HBEC3-p53KD-K-Ras^V12^ cells with intact BRMS1 expression (Fig. 3A). These results are consistent with the observation that the loss of BRMS1 results in an elongated cell shape and enhanced cell motility. Importantly, re-expression of BRMS1 following exposure the Ad-Cre significantly increased the shape factor score in HBEC3-p53KD-K-Ras^V12^ cells (0.60 vs. 0.20, p<0.05) supporting a partial reversion to a more epithelial phenotype associated with decreased motility. These data are consistent with data shown in Figure 2, where re-expression of BRMS1 in HBEC3-p53KD-K-Ras^V12^ -BRMS1 KD cells inhibited cell migration and invasion capability. These data suggest that the loss of BRMS1 leads to an elongated cell morphology resulting from F-actin reorganization and that this morphologic change is associated with increased cell motility.

In addition to the changes in actin reorganization and cell shape we found that BRMS1-KD cells displayed more defined leading edges with distinct lagging tails that were not present following BRMS1 re-expression. To examine the impact of *BRMS1* knock-down we measured the span of the angle of the leading edges of these cells to establish scores indicative of the directional leading edges. HBEC3-p53KD-K-Ras^V12^ cells expressing shRNA *BRMS1* had significantly smaller angles than the control cells (60 vs. 175, * p<0.05) and had distinct lagging tails, suggesting a more directional leading edge (Fig. 3B). As shown in representative images in Figs. 3B and C, the knockdown of *BRMS1* caused redistribution of paxillin with more paxillin accumulation at the leading edge of cell. This effect on paxillin was reversed as shown in additional images following re-expression of BRMS1 (Fig. S2D). Collectively, data shown in Figure 3 demonstrates that BRMS1 is required to maintain overall cell morphology by regulating changes in F-actin re-organization and paxillin accumulation at the leading edge of the lamellapodium.

### BRMS1 KD increases migration and invasion in NSCLC cell lines

**Figure 4:**
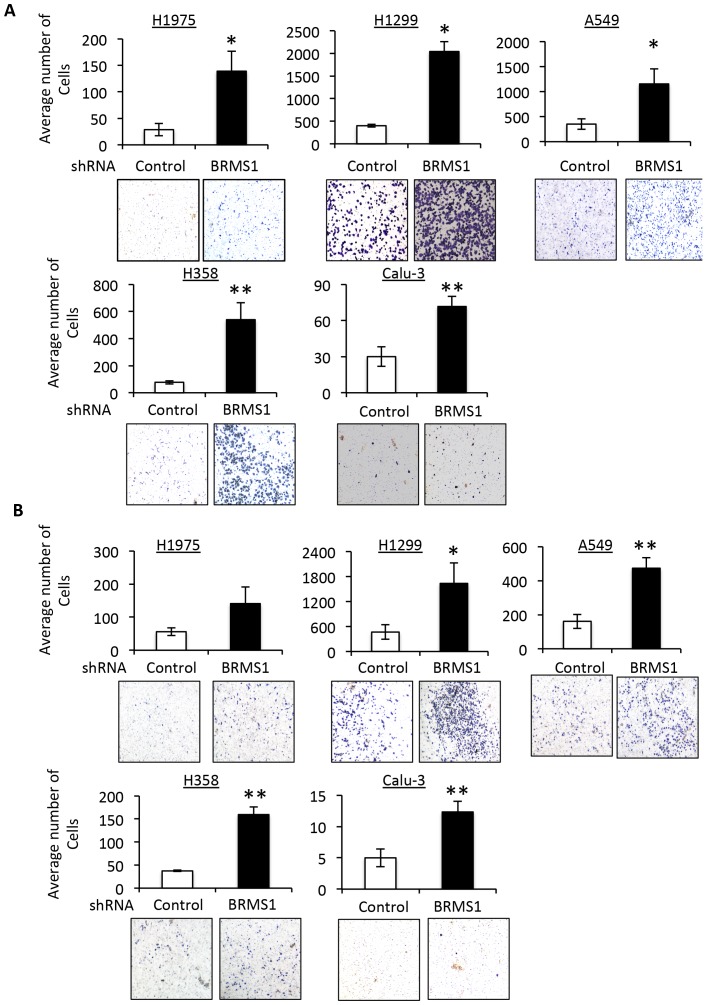
BRMS1 KD enhances migration and invasion of NSCLC cells. A) The indicated NSCLC cells were infected with lentiviruses encoding shRNA BRMS1 or shRNA control. Migration assays were performed. * p≤0.05 and ** p≤0.01 compared to control in each cell lines. B) The indicated NSCLC cells were infected with lentiviruses encoding shRNA BRMS1 or shRNA control. Invasion assays were performed. * p≤0.05 and ** p≤0.01 compared to control in each cell lines.

While we have shown that BRMS1 plays an important role in regulating migration and invasion of HBEC3 cells with the specific genetic makeup of p53 KD and oncogenic K-Ras^V12^ mutant, we sought to examine how BRMS1 affects the migration and invasion potential of NSCLC cells with similar oncogenic backgrounds. To address this, we knocked down BRMS1 in four NSCLC cell lines with different p53 and K-Ras status (H1975: p53 WT/K-Ras WT; H1299: p53 null/K-Ras WT; A549: p53 WT/K-Ras^S12^, H358: p53 null/K-Ras^C12^) and a non-migrating NSCLC cells, Calu-3 (p53 mut/K-Ras WT) (Fig. S3) (p53 status: http://p53.free.fr/Database/Cancer_cell_lines/NSCLC.html) [24]. Although these NSCLC control cell lines showed various capabilities to migrate and invade *in vitro*, BRMS1KD significantly promoted migration and invasion in almost all tested NSCLC cell lines (Figs. 4A and B).

## Discussion

The specific contributions and mechanisms through which BRMS1 impacts tumor cell migration and invasion remain unclear. To examine the specific role of BRMS1 in cell migration we chose to use the immortalized, non-transformed HBEC3 cells. HBEC3 cells were derived from the central airways of an adult lung [25]. Unlike cancer cell lines, these HBEC cells have a relative “normal” genetic background and provide an excellent model to study specific genetic alterations commonly seen in lung cancer [17].

In this study, we show that HBEC cells expressing p53 and K-Ras driver mutations but with preserved BRMS1 expression have limited cell migration. Following the loss of BRMS1, cells with p53 KD-K-Ras^V12^ mutations acquire the ability to migrate and invade. Importantly, re-expression of BRMS1 significantly attenuates this *BRMS1*-KD-induced cell migration. This study adds to the known mechanisms of action for BRMS1 and is the first to demonstrate in a pre-malignant genomic background the BRMS1-specific contributions to cell migration and invasion. It is important to note that introduction of *BRMS1*-KD into HBEC3 control cells does not promote cell migration or invasion, suggesting that the p53 and K-Ras driver mutations are a prerequisite for *BRMS1*-KD-induced cell motility and invasion.

Cell migration is a fundamental process in early morphogenesis and cancer metastasis [26]. Migration involves amoeboid movement with cycles of protrusion, adhesion, contraction and retraction [27]. Following F-actin re-distribution the moving cell becomes elongated and filopodium and lamellapodium are formed to dynamically interact with extracellular matrix (ECM) [28]. Contraction of actomyosin propels the cell forward, and in a final stage the tail at the trailing edge is retracted. We observed a migratory phenotype following the knockdown of *BRMS1* in our defined oncogenic background as evidenced by changes in the actin cytoskeletal network resulting in elongated cell shape and a significantly decreased cell shape factor score. We also observed that the migratory cell shape and actin cytoskeletal network correlates with the formation of distinct leading edge of migrating cells. Following the loss of *BRMS1* a more distinct and directional leading edge accompanied by lagging tails was observed which is consistent with increased cell motility and migration. In addition, *BRMS1*-KD resulted in the accumulation of paxillin in the leading edge of cells. The accumulation of paxillin controls dynamics of lamellapodia extension and efficient directional cell migration and invasion [29]. These observations confirm reports that overexpression of BRMS1 reduces cellular adhesion to ECM [30]. Therefore, preservation of BRMS1 levels helps maintain an epithelial cell morphology and inhibits cell motility via regulation of actin arrangement and paxillin distribution - both of which support the function of BRMS1 as a metastasis suppressor.

An interesting observation in our study is that the re-expression of BRMS1 does not completely reverse the phenotypic alterations observed following *BRMS1* knockdown. Possible explanations for this observation include an inefficiency of our loxP expression construct or more likely that loss of BRMS1 induces an irreversible mesenchymal phenotype perhaps through epigenetic modifications [31]. Although epithelial cells can undergo either reversible or irreversible mesenchymal changes, conversion to an oncogenic mesenchymal phenotype is typically considered “complete and irreversible” [32]. For example, transforming growth factor (TGF-β) induces the non-histone chromatin-binding protein HMGA2 which results in irreversible EMT changes in mammary epithelial cells [33], [34]. Although the mechanisms governing the irreversible mesenchymal state in cancer are unclear, oncogenic EMT is considered to be indicative of permanent reprogramming of cellular behaviors that are intrinsic to malignant cells [32].

While BRMS1 KD induction of both migration and invasion occurs in HBEC3 cells with a genomic background of p53 KD and oncogenic K-Ras^V12^, we observed that BRMS1 KD enhances migration and invasion in almost all tested NSCLC cell lines, regardless of the status of *p53* and *K-Ras*. Although mutations of *p53* and *K-Ras* genes are commonly observed in NSCLC, numerous other driver mutations have been identified, such as *EGFR, ALK, ERBB2, and BRAF* [35]. In addition, multiple transcriptional dysregulation and aberrant epigenetic modifications also greatly contribute to the development of NSCLC [36], [37]. It is difficult to identify a cancer cell line that possesses one or two oncogenic mutations, amplifications or deletions. For example, Calu-3 cells not only harbor mutations in *p53* and *CDK2A*, but have constitutively active ERBB2 [38]. As a transcriptional co-repressor and E3 ligase [8], [15], it is plausible that BRMS1, affects tumor metastasis involving a variety of cell signaling pathways in cancer cells.

In this study, we did not observe tumorigenesis in our *in vivo* p53KD-K-Ras^v12^ HBEC3 xenograft model. This is not surprising in that BRMS1 is a metastasis suppressor and has never been shown to be required for tumor initiation or growth. We cannot unequivocally exclude the possibility that further differentiation is required for HBEC cells to be converted to cancer given their recently described stem-ness features [25]. Evidence supporting this hypothesis was recently supplied by Soto et al, who found that introducing c-Myc, an important oncogene involving cell differentiation [39], into p53KD-K-Ras^v12^ HBEC cells is sufficient to induce full tumorigenic conversion [40].

In summary, using HBEC cells with known oncogenic driver mutations as a model of lung cancer progression we show BRMS1 to be an important regulator of cell migration and invasion. Furthermore, we identified that loss of BRMS1 results in increased cell motility, cytoskeletal reorganization, and paxillin redistribution – all supportive of a migratory and mesenchymal phenotype. Re-expression of BRMS1 partially rescues these effects; however it is incomplete, suggesting that loss of BRMS1 results in an irreversible mesenchymal state in premalignant epithelial cells.

## Supporting Information

Figure S1 **BRMS1 KD fails to increase cell migration and invasion in HBEC3 cells with p53KD or oncogenic K-Ras.** A) Western blots indicate the protein levels of p53, K-Ras and BRMS1 in HBEC3 cells with indicated genetic makeups. Actin was detected as a loading control. B) HBEC3-p53KD and HBEC3-K-Rasv12 cells were infected lentiviruses encoding shRNA BRMS1 or shRNA control. The protein levels of BRMS1 were probed by immunoblots. Actin is used as a loading control. C) HBEC3-p53KD and HBEC3-K-Rasv12 cells were infected lentiviruses encoding shRNA BRMS1 or shRNA control. Migration assays were performed. * p<0.05 compared to control in each cell line. D) HBEC3-p53KD and HBEC3-K-Rasv12 cells were infected lentiviruses encoding shRNA BRMS1 or shRNA control. Invasion assays were performed.
(PDF)

Figure S2 **BRMS1 KD regulates actin arrangement and paxillin distribution in HBEC3-p53KD-K-Ras^v12^ cells, but not HBEC3 control cells.** A) Representative images of HBEC3-p53KD-K-Ras^v12^ cells +/-BRMS1 KD with and without Ad-Cre recombinase treatment, stained with Rhodamine-Palloidin to visualize F-actin. B) Representative images of HBEC3 cells +/-BRMS1 KD, stained with Rhodamine-Palloidin to visualize F-actin. C) HBEC3-p53KD-K-Ras^v12^ Control and BRMS1 KD cells were treated with or without Ad-Cre. F-actin was visualized by Rhodamine-Palloidin staining. GFP expression is shown as a control for the efficiency of Ad-Cre. The white arrows indicate stress fibers and the yellow arrows indicate the lamellipodia and filipodia. D) HBEC3-p53KD-K-Ras^v12^ Control and BRMS1 KD cells were treated with or without Ad-Cre. Paxillin was visualized by Rhodamine-Palloidin staining. GFP expression is shown as a control for the efficiency of Ad-Cre. The white arrows indicate stress fibers and the yellow arrows indicate the lamellipodia and filipodia.
(PDF)

Figure S3 **BRMS1 is efficiently knocked down in NSCLC cell lines**. The indicated NSCLC cells were infected lentiviruses encoding shRNA BRMS1 or shRNA control. The protein levels of BRMS1 were probed by immunoblots. Actin is used as a loading control.
(PDF)
