# Supplementary figures and images for: Inhibition of Breast Cancer Metastasis Suppressor 1 Promotes a Mesenchymal Phenotype in Lung Epithelial Cells That Express Oncogenic K-RasV12 and Loss of p53
Source: PLoS One. 2014 Apr 24;9(4):e95869. doi: 10.1371/journal.pone.0095869 (PMC3999110; doi:10.1371/journal.pone.0095869)

**Fig. S1**

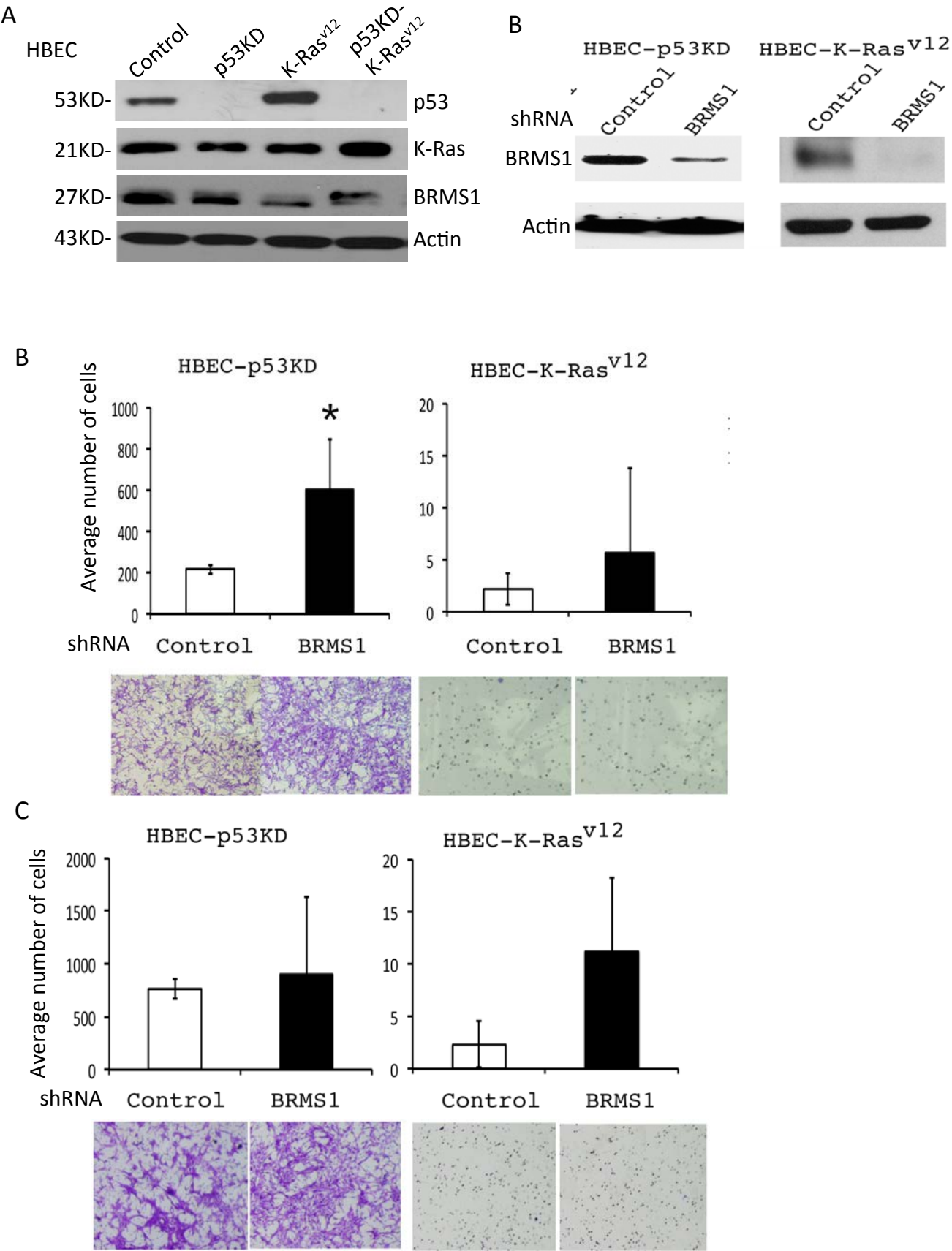

Supplement: Figure S1 — BRMS1 KD fails to increase cell migration and invasion in HBEC3 cells with p53KD or oncogenic K-Ras. A) Western blots indicate the protein levels of p53, K-Ras and BRMS1 in HBEC3 cells with indicated genetic makeups. Actin was detected as a loading control. B) HBEC3-p53KD and HBEC3-K-Rasv12 cells were infected lentiviruses encoding shRNA BRMS1 or shRNA control. The protein levels of BRMS1 were probed by immunoblots. Actin is used as a loading control. C) HBEC3-p53KD and HBEC3-K-Rasv12 cells were infected lentiviruses encoding shRNA BRMS1 or shRNA control. Migration assays were performed. * p<0.05 compared to control in each cell line. D) HBEC3-p53KD and HBEC3-K-Rasv12 cells were infected lentiviruses encoding shRNA BRMS1 or shRNA control. Invasion assays were performed. (PDF) [file pone.0095869.s001.pdf]

**Fig. S2**

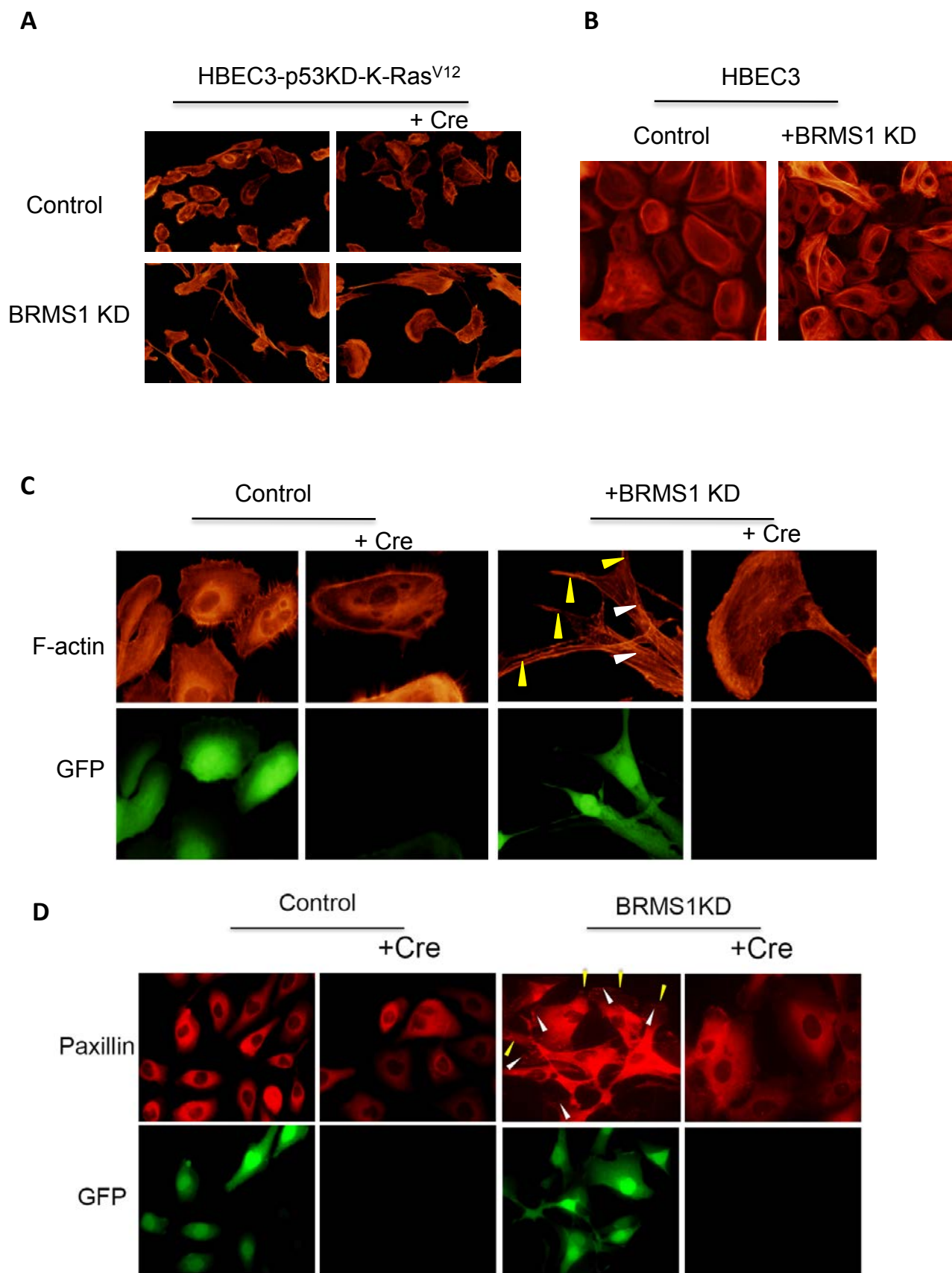

Supplement: Figure S2 — BRMS1 KD regulates actin arrangement and paxillin distribution in HBEC3-p53KD-K-Rasv12 cells, but not HBEC3 control cells. A) Representative images of HBEC3-p53KD-K-Rasv12 cells +/-BRMS1 KD with and without Ad-Cre recombinase treatment, stained with Rhodamine-Palloidin to visualize F-actin. B) Representative images of HBEC3 cells +/-BRMS1 KD, stained with Rhodamine-Palloidin to visualize F-actin. C) HBEC3-p53KD-K-Rasv12 Control and BRMS1 KD cells were treated with or without Ad-Cre. F-actin was visualized by Rhodamine-Palloidin staining. GFP expression is shown as a control for the efficiency of Ad-Cre. The white arrows indicate stress fibers and the yellow arrows indicate the lamellipodia and filipodia. D) HBEC3-p53KD-K-Rasv12 Control and BRMS1 KD cells were treated with or without Ad-Cre. Paxillin was visualized by Rhodamine-Palloidin staining. GFP expression is shown as a control for the efficiency of Ad-Cre. The white arrows indicate stress fibers and the yellow arrows indicate the lamellipodia and filipodia. (PDF) [file pone.0095869.s002.pdf]

Fig. S3

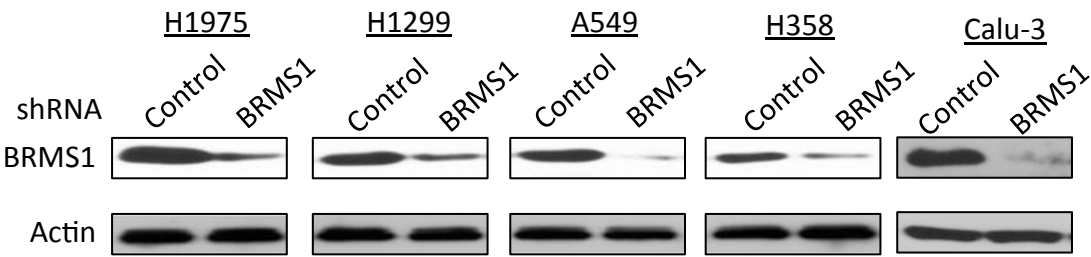

Supplement: Figure S3 — BRMS1 is efficiently knocked down in NSCLC cell lines. The indicated NSCLC cells were infected lentiviruses encoding shRNA BRMS1 or shRNA control. The protein levels of BRMS1 were probed by immunoblots. Actin is used as a loading control. (PDF) [file pone.0095869.s003.pdf]
